# Employing the TAM in predicting the use of online learning during and beyond the COVID-19 pandemic

**DOI:** 10.3389/fpsyg.2023.1104653

**Published:** 2023-02-17

**Authors:** Tahereh Zobeidi, Seyedeh Bahar Homayoon, Masoud Yazdanpanah, Nadejda Komendantova, Laura A. Warner

**Affiliations:** ^1^Cooperation and Transformative Group, International Institute for Applied Systems Analysis (IIASA), Laxenburg, Austria; ^2^Department of Agricultural Extension and Education, Agricultural Sciences and Natural Resources University of Khuzestan, Mollasani, Iran; ^3^Department of Agricultural Education and Communication, Institute of Food & Agricultural Sciences, University of Florida, Gainesville, FL, United States

**Keywords:** internet anxiety, internet self-efficacy, psychology, agricultural education, TAM

## Abstract

Online learning systems have become an applied solution for delivering educational content, especially in developing countries, since the start of the COVID-19 pandemic. The present study is designed to identify the factors influencing the behavioral intention of agricultural students at universities in Iran to use online learning systems in the future. This research uses an extended model in which the constructs of Internet self-efficacy, Internet anxiety, and output quality are integrated into the technology acceptance model (TAM). Data analysis was performed using the SmartPLS technique. The analyses showed the proposed model to be strong in terms of predicting the attitude to online learning and the intention to use it. The extended TAM model fit the data well and predicted 74% of the intention variance. Our findings show attitude and perceived usefulness to have directly affected intention. Output quality and Internet self-efficacy indirectly affected attitude and intention. Research findings can help with the design of educational policies and programs to facilitate education and improve student academic performance.

## 1. Introduction

COVID-19 first appeared in December 2019 in Wuhan, China. Based on World Health Organization (WHO) statistics, the deadly disease was infecting millions and leaving thousands dead (World Health Organisation, 2021; Hallaj et al., 2022; Moradhaseli et al., 2022). As a response to the COVID-19 crisis, governments around the world have been implementing public policies such as social distancing, isolation, and quarantine (Anderson et al., 2020; Amghani et al., 2022). Many Asian and European countries have shut down large communities, including educational institutions, to combat this “invisible” disease (Sahu, 2020). In these circumstances, universities have turned to advances in information technology as a possible means of overcoming the difficulties being encountered (Raza et al., 2021). COVID-19 has been unprecedented in changing the way students are taught around the world within a short period of time (Chung et al., 2020). Many universities have started to use online learning as a way of ensuring continuity of education (Chung et al., 2020), and have moved rapidly from face-to-face courses and programs to delivery of educational content online (Gewin, 2020). Iran is one of the countries most affected by the outbreak of COVID-19. To prevent the transmission of the virus, the Iranian government closed educational institutions on 5 March 2020 (Taghizadeh et al., 2021).

Online learning is an established, effective, and influential learning medium in many educational organizations and institutions (Lee et al., 2020). Online learning, an advanced e-learning method, includes training, coaching, information, and any learning content that is presented digitally or electronically (Fallon and Brown, 2002). Online learning programs, such as chat meetings, posts, and emails, facilitate communication between teachers and students in a variety of ways (Romanov and Nevgi, 2006). In Iran, the University of Tehran, with the establishment of the Electronic Education Center in 2002, is the first university that started studying and planning online education, but the first collegiate virtual course was held in Shiraz University in 2003, and subsequently in the universities of science and technology, Khajeh Nasir al-Din Toosi University of Technology, and Amir Kabir. Most of the large public universities in Iran have established e-learning centers so that the quantitative growth of students in e-courses has been increasing from 235 in 2013 to 19,000 in 2013 (Sharifi et al., 2019). It is argued that COVID-19 accelerates the digital transition in the world, which provides solutions regardless of the physical world (Arpaci et al., 2022). In Iran, the Covid-19 pandemic led to the flourishing of some capabilities in the country, including the spread and prosperity of virtual education throughout the country, so that virtual education in Iran has entered a new phase and more attention has been paid to virtual education (Yaghoubi Farani et al., 2019). On the other hand, those in charge have also become more aware of the importance of distance education and e-learning education (Ghafourifard, 2020). Online learning provides learning opportunities anytime and anywhere (Zamani-Miandashti and Ataei, 2015; Joosten and Cusatis, 2020); it focuses on learners’ needs and preferences and also activates critical thinking on the part of students. This type of learning environment also increases the efficiency and success of learning facilities by improving communication between students and educators (Idris and Osman, 2015). These benefits, together with the increasing demands being placed on higher education establishments regarding the admission of new students and how their needs can be met, are the justification for online education (Kim et al., 2005). The flexible use of online media does, however, require students to have skills such as knowledge of technology use, time and organization management, and interaction using online technologies (Joosten and Cusatis, 2020). Educational institutions are thus investing in information systems to offer increased access to education and to improve students’ self-efficacy, knowledge production, cost-effectiveness, together with learner flexibility and engagement (Sinclair et al., 2016). However, if the e-learning system is not used properly, it is difficult to derive its full benefits (Almaiah and Al-Khasawneh, 2020). Such could also lead to the failure of the education system and reduce universities’ return on investment (Naveed et al., 2017). In fact, the success of the e-learning system relies on the intention and readiness of students to use it (Shawai and Almaiah, 2018; Almaiah and Alismaiel; Al-Adwan et al., 2021). Research into online learning is still in its infancy (Tarhini et al., 2017; Almaiah and Alamri, 2018). Participation in e-learning needs more in-depth study so that the requirements of university students can be better understood: this will ultimately lead to a more successful e-learning system (Alksasbeh et al., 2019).

Despite the importance of examining students’ intention to use online learning systems, there are few literature reviews on the intention to use – and the actual use of – online learning (Mouakket and Bettayeb, 2016; Al-Adwan et al., 2021). As, to our knowledge, there is no research that examines the intention of Iranian students to use online learning and its predictors, the present study aims to address these two issues critical gaps in knowledge.

Many theoretical frameworks have been applied to cognitive aspects of the use of technology in education (Teo et al., 2018). Theoretical models, such as the task–technology fit model (Venkatesh et al., 2003), social cognitive model (Bandura, 1986), the theory of planned behavior (Ajzen and Fishbein, 1975), and the technology acceptance model (TAM) (Venkatesh and Davis, 2000) have been applied to study students’ attitudes, perceptions, and behaviors regarding online learning. Of these, TAM is reported to be the best broadly functional model in the arena of social sciences (Teo et al., 2018). Although TAM has been lately disapproved for being an obsolete model, the bibliometric analysis study of (Al-Emran and Granić, 2021) specified that the number of research’s on TAM and its applications are on the rise, providing indication the model is still valid across several applications and fields.

Several years after the advent of TAM, this model was expanded, with various external variables being added to it (Rahmawati, 2019). The expansion was critical to TAM, as TAM did not then pay enough attention to individual characteristics (Agarwal and Prasad, 1999; McMaster and Wastell, 2005; Cheng, 2011; Khayun and Ractham, 2011). Scientists have conducted studies with the extended TAM model, using differing variables in different countries and different fields of e-learning and especially during and beyond Covid-19 (Sukendro et al., 2020; Al-Adwan et al., 2021, 2022; Aboagye et al., 2021; Mailizar et al., 2021; Al-Nuaimi et al., 2022; Han and Sa, 2022). Al-Adwan et al. (2021, 2022) focused on quality factors of e-learning systems, and self-directed learning in the extended TAM. Han and Sa (2022) used educational satisfaction instead of attitude to explain acceptance intention of online education. Sukendro et al., (2020) also added facilating conditions to the TAM. In addition, facilitating conditions and major barriers are effective in the adoption of online learning (Sukendro et al., 2020). One of these conditions is the availability of adequate internet speed, especially in developing countries. Iran’s situation in terms of internet speed has been inadequate. Among 211 countries in the world, Iran ranks 117th in terms of internet speed (Lahordi, 2021). Therefore, concern about the state of the Internet is considered one of the most important factors that can affect the acceptance of online education in Iran. To our best knowledge, there is not any study in Iran which considered the perceived anxiety and internet self-efficacy along with the dimensions of the outputs quality. The novelty of this study is considering the dimension of internet anxiety and internet self-efficacy considering low Internet speed in Iran. Precisely, this study’s contribution is two-fold. First, the study goals to discover agricultural students’ intention to adopt online-learning and the conditional factors including internet anexity and internet self-efficacy that affect their intention during and beyond the COVID-19 disaster. Second, the study pursues to inspect online learning performances based on empirical data from a developing country, Iran.

The contribution of this study extends to university policy makers and educators who can gain deeper insight and understanding of student acceptance of online learning technology beyond Covid-19, leading to better e-learning policy development.

This study uses the TAM, extended by specific constructs, to predict the intention to use online learning beyond Covid-19. To achieve this general goal, we pursued the following specific objectives:

- Use of TAM in predicting students’ intention to use online learning.
- Use of the expanded TAM to improve the explanatory power of the intention to use online learning.

The paper is organized as follows. Section 2 outlines the literature review with the main components of the theoretical framework and its expanded version. In this section, we explain TAM as the underlying framework to examine psychological factors affecting students’ adoption of online learning. Specifically, we corporate the TAM with the constructs of output quality, perceived internet anxiety, and internet self-efficacy, which has been explained in this section. Section 3 discusses the methodology including case study features, constructs measurements, and data analysis. Section 4 presents the results, section 5 discusses the finding, and section 6 discusses the theoretical and practical implications for the development use of online education systems in higher education. Finally, the conclusion and limitations are discussed in section 7.

## 2. Literature review

### 2.1. Technology acceptance model (TAM)

TAM, which was introduced by Fred D. Davis in 1986, is considered to be the greatest influential theory describing the acceptance of information systems (Lee et al., 2003). A well-known theory on the use and prediction of information technology acceptance, its capability has been validated by several studies in the domain of e-learning (Lee et al., 2011; Padilla-Meléndez et al., 2013; Lee et al., 2014; Tarhini et al., 2014; Wu and Zhang, 2014; Al-Gahtani, 2016; Rahman et al., 2016). The model is rooted in the Theory of Reasoned Action and Theory of Planned Behavior which were proposed by Ajzen and Fishbein (1980), Sarcheshmeh et al. (2018), and Ajzen (1985), respectively, under the theories of Social Psychology (Al-Fraihat et al., 2020). TAM has been formed as an analytical, and influential model for amplification and forecasting performance in decision-making as well as acceptance of using a particular technology (Savari et al., 2021). Based on the TAM, the current and future use of online learning systems is determined by two variables, perceived usefulness (PU) and perceived ease of use (PEOU) (Davis, 1993). In the TAM, PU refers to a person’s belief that the use of technology improves their work performance (Davis, 1989), and PEOU is defined as the degree to which one believes technology is easy to use and free from effort (Davis, 1989). Intention is defined as a cognitive process, based on people’s readiness to perform a target behavior (Abbasi et al., 2011).

The literature on information systems indicates that higher PEOU leads to higher PU. Indeed, when students perceive using the Internet is simple—they think it is very useful (Ebrahimi et al., 2018). This positive and significant relationship between the two variables is also supported by research in the field of e-learning (Lee et al., 2009; Faqih, 2013; Ibili et al., 2019; Demoulin and Coussement, 2020).

According to TAM, PU and PEOU affect people’s attitudes toward using technology. These relationships are supported by different studies in mobile technology (Briz-Ponce et al., 2017), learning management systems (Revythi and Tselios, 2019), cloud services (Huang, 2016), and e-learning (Al-Rahmi et al., 2019; Salloum et al., 2019). Arpaci (2017) also found PU and PEOU affect attitudes toward using distance education tools and systems.

Attitude refers to an individual’s assessment (favorable/unfavorable and or positive/negative feelings) regarding the performance of a given behavior (Shahangian et al., 2021; Valizadeh et al., 2021; Ataei et al., 2021a,b). According to research (Sukendro et al., 2020), attitudes toward the use of online education during Covid-19 could affect a person’s behavioral intentions to use online education.

Buabeng-Andoh (2021) examined the behavior of students in developing countries and investigated their behavioral intention regarding the use of mobile learning, showing that attitude has a great impact on behavioral intentions: if students have a positive attitude toward technology, they are more likely to use it. In particular, research conducted in developing countries, such as Chang et al. (2017) in Azerbaijan, Hussein (2017) in Malaysia, and Hanif et al. (2018) in Pakistan, found attitude to be a significant predictor of the intention to use learning systems. Research has shown that PU also has a direct and positive effect not only on attitude but also on behavioral intentions. Studies have found PU to have a positive effect of on intention toward using electronical and multimedia online learning systems (Al-Rahmi et al., 2019; Hariguna, 2019; Salloum et al., 2019; Teo et al., 2019). The study results of Al-Adwan (2020) showed students’ behavioral intention to adopt massive open online courses is positively affected by the PEOU and PU. Al-Emran et al. (2020) also found that PEOU and PU have significant effects on students’ behavioral intention to use smartwatches for educational purposes.

### 2.2. Expanding the technology acceptance model

TAM focuses primarily on technology-related features but ignores other key non-technological factors, such as individual traits, that play a critical role in the use of technology. In the current research, therefore, steps have been taken to expand the TAM model using two constructs, “Internet self-efficacy” and “Internet anxiety,” which are based on individual characteristics, plus another construct, namely, “output quality,” which also plays a key role in using online learning systems.

Individual characteristics are significantly diverse. A vital individual variable in terms of technology use is self-efficacy (Mahdavian et al., 2016). The first individual construct added to TAM was Internet self-efficacy (Cheng, 2011; Zhao et al., 2011). Self-efficacy is defined as a person’s belief they can successfully carry out the necessary actions to produce the desired results in a certain context (Yazdanpanah et al., 2020; Karataş et al., 2022; Savari et al., 2022; Tajeri Moghadam et al., 2022) and Internet self-efficacy refers to a person’s judgment regarding their ability to successfully carry out actions on the Internet (Compeau and Higgins, 1995; Eastin and LaRose, 2000; Yuen and Ma, 2008). Internet self-efficacy is an important predictor in TAM (Venkatesh and Bala, 2008; Chung et al., 2010; Chang and Im, 2014; Tsai et al., 2016). Studies (Park, 2009; Isaac et al., 2017) have demonstrated significant effects of Internet self-efficacy on PU and PEOU.

The second individual construct added to the model is Internet anxiety; anxiety regarding technology is linked to the person’s inner self-control (Hernández-García and Iglesias-Pradas, 2012). This variable is also related to the undesirable emotions a person can experience when they work with technology (Sánchez-Prieto et al., 2017). Internet anxiety itself comes from the construct of computer anxiety. Computer anxiety in the education field is defined as restlessness, apprehensiveness, or fear of present or future computer use (Igbaria and Parasuraman, 1989) in education (Sánchez-Prieto et al., 2017). Alenezi and Karim (2010) argue that computer anxiety plays a major role in the acceptance of e-learning in higher education organizations. Studies have indicated that anxiety is related to avoidance of e-learning systems or technologies or to using them infrequently (Chen and Tseng, 2012; Park et al., 2012; Purnomo and Lee, 2013). More anxious. students are less likely to use e-learning systems (Al-Alak and Alnawas, 2011). In particular, studies have supported the negative effect of computer anxiety on PEOU (Karaali et al., 2011; Chen and Tseng, 2012; Park et al., 2012; Ali et al., 2013; Calisir et al., 2014; Al-Gahtani, 2016). Research has also confirmed that anxiety has a negative effect on PU (Park et al., 2012; Purnomo and Lee, 2013). The more anxious students are, the less useful they perceive a computer to be.

**Figure 1:**
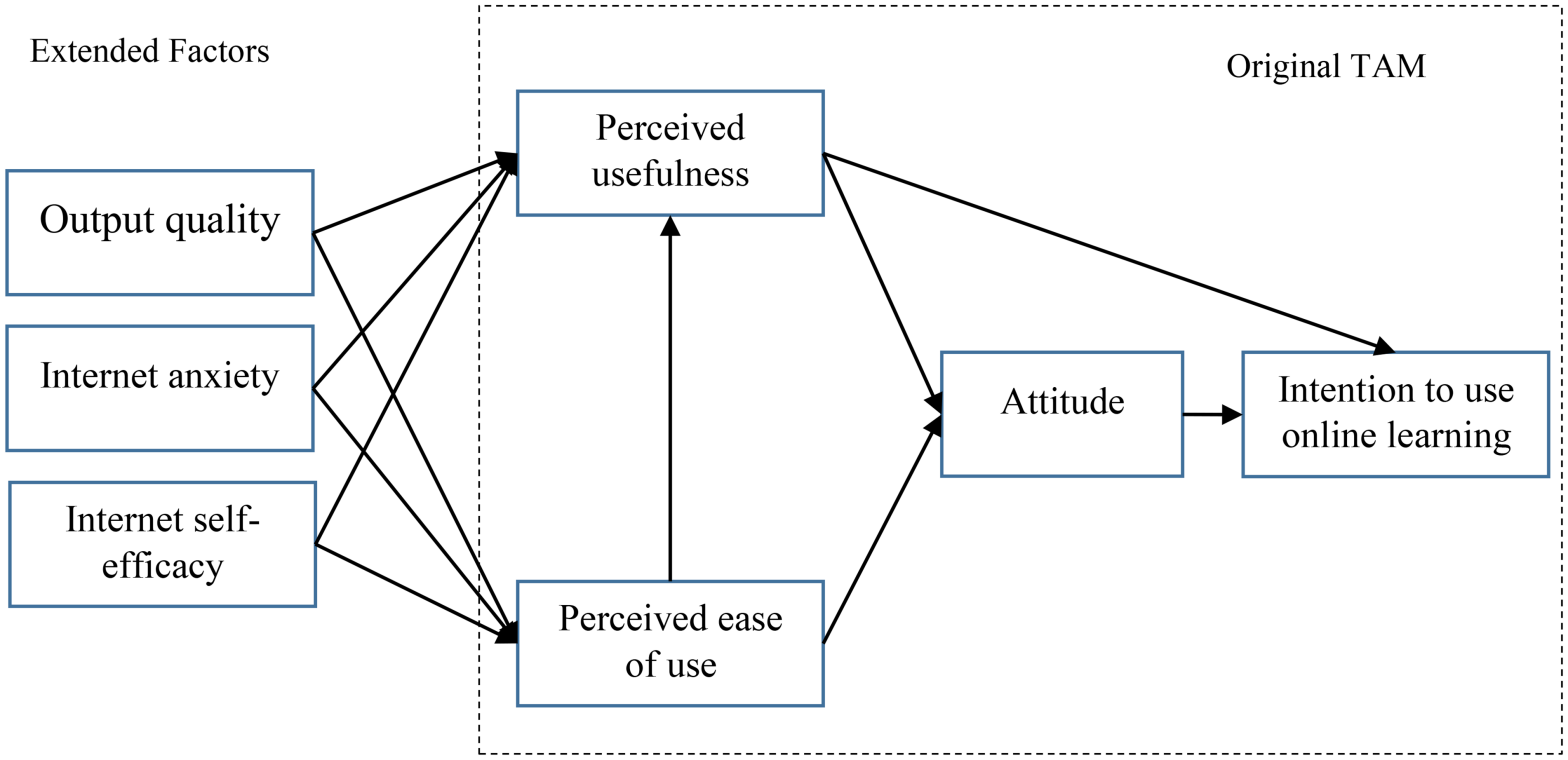
Extended TAM.

The third construct added to the TAM is output quality. Output quality refers to the degree to which a person thinks or believes a system works satisfactorily and is defined as the level of positive effects of a person’s use of an information system (Venkatesh and Davis, 2000). Several studies indicated that output quality influences PU and PEOU (Davis et al., 1992; Venkatesh and Davis, 2000; Lee et al., 2018). Meanwhile Ji et al. (2019) argue that the quality of outputs is essential for users to continue using online learning programs. Figure 1 shows the research extended TAM employed by the present study which was drawn based on a vast literature review.

## 3. Methodology

### 3.1. Participants and sample

The present research was performed using a cross-sectional study. The statistical population of the study consisted of agricultural students in Iran. According to the Institute for Research and Planning in Higher Education of Iran, 115,261 students are studying different fields of agriculture and at different academic levels in the 2020–2021 academic year. Data collection was done in the summer of 2020 after holding of the second virtual education course since the COVID-19 outbreak. An online survey was designed, and a convenience sampling method was used to distribute it online among agricultural students at Iranian universities (*N* = 115,261). Various tools including information sharing platforms, such as WhatsApp and Telegram, forums, and other sites were used to distribute the questionnaire. Finally, 480 completed questionnaires were collected; the response rate was 51%. Based on Cochran’s equation, 383 samples are enough for the research target population, however, in this study, more samples were selected to ensure the findings. Descriptive statistics on students’ individual characteristics showed that the average age of students was about 24.5 years (*SD* = 6.46). The minimum age was 18 and the maximum was 54. The mean family size was 4.71 (*SD* = 1.66). Of 480 respondents, 69.6% were women and 30.4% were men. Of all respondents, 365 were undergraduate students (76.5%), 13.6% were master’s students, and 9.9% were doctoral students. Of the respondents, 84.1% had no experience of using virtual training prior to the COVID-19 outbreak, while 15.9% had participated in online classes.

### 3.2. Questionnaire development

In this study, a structured two-part questionnaire was applied to examine the research framework. The first section consisted of questions related to the demographic information of the participants. The second measured the variables of the research framework. Items related to all constructs were quantified using 5-point Likert scales. The response choices for all items except Internet self-efficacy ranged from “strongly disagree” (1) to “strongly agree” (5). For the Internet self-efficacy items, students were requested to specify how confident they were in their ability to use the Internet for each of the alternatives and to indicate their responses on a scale from “very low” (1) to “very high” (5).

To ensure the validity of the questionnaire, a group of experts from different agricultural and environment education fields edited and approved the questions. Cronbach’s alpha reliability coefficients obtained through a pilot study involving 30 students indicated reliability from very good to excellent, generally 0.81–0.912 for all scales.

The questionnaire consisted of 30 total items that addressed the factors included in the research model; some items with low item-to-item correlation were omitted in the statistical analysis and 25 were used in the final analysis. Details of the scale of the items and the literature source for each construct are provided in Table 1.

**Table 1:** Items of the extended TAM model and their sources.

Items	Source
Internet anxiety	
I get confused when working with the internet	Calisir et al. (2014) and Sánchez-Prieto et al. (2017)
I get anxious when I think about taking an online course	
I get nervous when I am asked to participate in online discussions	
I’m worried about enrolling in online courses	
I avoid working on the internet	
I get anxious when I need to use online resources	
I get nervous about getting lost in virtual space	
Intention	
I plan to take virtual classroom systems courses in the future if held at the university	Tao et al. (2019), Park (2009) and Davis (1989)
I intend to be an active user in an online-learning system	
I recommend that some courses be held virtually after COVID-19	
Internet self-efficacy	
How confident are you about your ability in each of the following?	Kuo (2010) and Kuo et al. (2014)
Troubleshooting Internet hardware	
Learning advanced skills in an online course	
Creating an online chat group if needed	
Understanding Internet hardware terms/words	
Attitude	
I think it was a good idea to attend the online classes	Sivo et al. (2018), Calisir et al. (2014), Park (2009) and Davis (1989)
Output quality	
The scientific quality of the online classes was the same as the face-to-face classes I had	Eom et al. (2006)
I learned as much from online classes as I did from the face-to-face version of the course	
The quality of the learning experience in online classes is better than in-class classes	
PEOU (PEOU)	
It was easy for me to learn how to use online classes	Sivo et al. (2018), Calisir et al. (2014), Tao et al. (2019), Park (2009), Davis (1989) and Pal and Vanijja (2020)
I think the process of using online classroom systems is clear and understandable	
Acquiring skills using online classroom systems is easy for me	
PU (PU)	
Online classes provided me with a valuable learning experience	Davis (1989) and Pal and Vanijja (2020)
Online classes minimize inequalities in education	
Assessing the success of online classes is a somewhat objective exercise	

### 3.3. Data analysis

We used structural equation modeling (SEM) to explore the associations among constructs. SEM, a path analysis with latent variables, is a method that is commonly used in the behavioral and social sciences to validate and test hypothetical or theoretical models (Yazdanpanah et al., 2021; Zobeidi et al., 2022). SEM technique contained of factor analysis and multiple regression analysis and was functional to assess the structural relationship between the measured and latent variables. SEM is a version of regression that includes a “measurement model” for some variables in general analysis. These variables, rather than being represented by a single-item, are represented by multiple items that are “weighted” in a manner similar to factor analysis. The benefit of SEM is that these thoughts are usually more consistent than single-item indexes (Zobeidi et al., 2021).

SEM is generally divided into covariance-based modeling (CB-SEM) and partial least squares (PLS-SEM). To select the most appropriate analysis technique, Kolmogorov–Smirnov and Shapiro–Wilk normality tests were used to evaluate the normality of the sample. The results indicate that the hypothesis of normal data distribution at the level of 1% is rejected. Moreover, kurtosis and skewness coefficients showed that the range of kurtosis coefficients was from −1.509 to −0.44 and the range of skewness coefficients from −0.093 to 0.567. To evaluate the model, because of the lack of sample distribution normality, the PLS-SEM technique was used with SmartPLS software version 3.2. We bootstrapped 2,000 samples to measure the significance of the path coefficients.

## 4. Results

### 4.1. Measurement model

To test the extended TAM, the PLS-SEM approach was used due to the non-normal distribution of data. PLS-SEM was performed using SmartPLS software, version 3.2. First, factor analysis was used to scan the reliability of items. As shown in Table 2, the factor loading of all items surpassed the acceptable threshold of 0.7 (Nunnally, 1978). The items thus had good reliability.

**Table 2:** Description of items.

	Mean	SD	Factor loading	*t* value	VIF
Internet anxiety	2.71	1.0315			
Anx1	2.49	1.173	0.751	28.859	2.064
Anx2	2.99	1.380	0.852	60.619	3.060
Anx3	2.94	1.309	0.840	48.339	2.730
Anx4	2.92	1.327	0.882	79.420	3.548
Anx5	2.24	1.174	0.717	28.511	1.795
Anx6	2.53	1.282	0.744	27.239	2.091
Anx7	2.94	1.366	0.776	30.470	2.091
Itention	2.67	1.339			
In1	2.58	1.421	0.945	160.071	4.114
In2	2.81	1.387	0.913	92.531	3.057
In3	2.62	1.542	0.899	73.964	2.773
Internet efficacy	2.77	0.923			
IE1	2.36	1.106	0.797	39.576	2.316
IE2	2.57	1.147	0.745	24.876	1.981
IE3	3.21	1.212	0.874	85.855	1.835
IE4	2.94	1.153	0.760	30.692	1.557
Attitude					
Att1	2.80	1.501	1	-	1.000
Output quality	2.24	1.218			
Q1	2.26	1.309	0.913	82.361	3.433
Q2	2.28	1.326	0.932	114.048	3.785
Q3	2.23	1.311	0.899	71.051	2.458
Perceived ease to use	2.93	1.253			
PEOU1	2.97	1.346	0.929	110.993	3.442
PEOU2	2.81	1.360	0.929	110.685	3.408
PEOU3	3.02	1.318	0.926	104.844	3.531
Perceived usefulness	2.61	1.187			
PU1	2.68	1.373	0.888	78.184	2.205
PU2	2.56	1.360	0.902	76.991	2.586
PU3	2.63	1.241	0.891	68.919	2.501

The convergent and discriminant validity of the model were also assessed. Composite reliability (CR) and average variance extracted (AVE) indices were applied to confirm convergent validity. Table 3 shows that all values were higher than 0.7 for CR and 0.5 for AVE, thus indicating convergence validity (Fornell and Larcker, 1981).

**Table 3:** Discriminant analysis and convergent validity.

	Output quality	Internet anxiety	Internet-efficacy	PU	PEOU	Attitude	Intention
Output quality	**0.917**						
Internet anxiety	−0.415	**0.797**					
Internet self-efficacy	0.406	−0.599	**0.796**				
PU	0.794	−0.402	0.456	**0.894**			
PEOU	0.645	−0.605	0.600	0.706	**0.928**		
Attitude	0.703	−0.404	0.458	0.755	0.687	**1**	
Intention	0.749	−0.439	0.465	0.783	0.732	0.824	**0.919**
Cronbach’s alpha	0.905	0.905	0.812	0.874	0.919	1	0.908
Composite reliability	0.94	0.924	0.873	0.922	0.949	1	0.942
Average variance extracted (AVE)	0.84	0.635	0.633	0.799	0.861	1	0.845

Bolded elements indicate the square root of AVE.

The Fornell–Larcker criterion was used to assess discriminant validity. Based on this criterion, discriminant validity confirmed when the variance of the variables of a model was higher than the shared variance of each variable with its items (Fornell and Bookstein, 1982). To calculate the discriminant validity, the square root of each construct was compared with the correlation between the variables. As Table 3 shows, all variables meet the Fornell–Larcker criterion.

In addition to the Fornell-Larcker criterion, HTMT method was used to check the discriminant validity. There are two methods on HTMT ratio and HTMT inference which used to evaluate discriminant validity with HTMT. The HTMT ratio method is to calculate the HTMT ratio through the application of the PLS algorithm from the original sample data. In this method, to approve the discriminant validity, the indices obtained from the HTMT ratio should be less than 0.85 or 9.0 (Hair et al., 2019). The second method is the HTMT inference method, which is obtained by analyzing the confidence intervals obtained from HTMT after the bootstrap process. To endorse the discriminant validity using HTMT inference, the confidence intervals obtained should not contain a value of 1. As Table 4 shows, HTMT scores in all relationships were less than 0.9. Also, HTMT inference did not show a value of 1 within the confidence interval of any of the relationships. Therefore, the results confirmed the existence of discriminant validity.

**Table 4:** Results of the HTMT.

	HTMT ratio						
	Output quality	Internet anxiety	Internet-efficacy	Perceived usefulness	Perceived ease to use	Attitude	Intention
Output quality							
Internet anxiety	0.44						
Internet self-efficacy	0.437	0.676					
Perceived usefulness	0.89	0.428	0.494				
Perceived ease to use	0.705	0.651	0.659	0.786			
Attitude	0.738	0.402	0.462	0.806	0.716		
Intention	0.825	0.459	0.493	0.877	0.799	0.865	
	**HTMT inference (2.5–97.5%)**						
Output quality							
Internet anxiety	0.348–0.527						
Internet self-efficacy	0.338–0.526	0.605–0.740					
Perceived usefulness	0.851–0.919	0.332–0.512	0.403–0.576				
Perceived ease to use	0.641–0.761	0.579–0.718	0.579–0.727	0.719–0.840			
Attitude	0.685–0.789	0.315–0.485	0.383–0.542	0.755–0.848	0.660–0.774		
Intention	0.775–0.862	0.369–0.539	0.408–0.578	0.835–0.915	0.737–0.849	0.821–0.904	

Furthermore, it is vital to inspect the presence of multicollinearity at this phase. Multicollinearity between independent constructs can significantly distort the interpretation of the consequences. Therefore, VIF “Variance inflation factor” valuation is directed to regulate the presence of multicollinearity. To designate the nonappearance of multicollinearity, the metric of VIF for each independent variable should be <5, however ideally, the VIF values should be close to 3 and lower (Hair et al., 2019). As Table 4 shows, the absence of multicollinearity is confirmed, as the VIF for all variables are far less than the cut-off threshold of 5.

### 4.2. Structural model

After confirming the reliability and validity of the measurement model, the structural model of extended TAM was run. The structural model estimates the percentage of variance predicted by the model for endogenous constructs using the coefficient of determination (*R*^2^); it also constructs cross-validated redundancy (*Q*^2^), and the magnitude of the direct and indirect effects of the constructs using path coefficient and f square size, and their significance.

The Q^2^ criterion was used to confirm the predictive relationship of the model through the Stone-Geisser test. According to Henseler et al. (2009), values of 0.02, 0.15, and 0.35 can be interpreted as low, medium, and strong predictive power, respectively. Chin (1998) points out that *R*^2^ above the threshold of 0.19, 0.33, and 0.67 are weak, moderate, and substantial, respectively.

Based on *R*^2^ and *Q*^2^ shown in Tables 5, 6, the extended TAM is a strong model for predicting intention. The proposed model can predict 74% of the variance in students’ intention to use online learning. This model also predicts about 62% of attitude variance through the effect of PU and PEOU. Moreover, 70 and 60% variance, respectively, of PU and PEOU changes, are predicted by the extended TAM. Therefore, the research model is a strong model in terms of predicting intention.

**Table 5:** Structural path analysis result.

Direct effect	*β*	Std. *β*	SD	*T* Statistics	*f* ^2^	*p* values	Results
Output quality - > PU	0.568	0.582	0.039	15.205	0.657	0.000	Supported
Output quality - > PEOU	0.424	0.425	0.036	11.006	0.354	0.000	Supported
Internet anxiety - > PU	0.080	0.087	0.036	2.481	0.014	0.013	Supported
Internet anxiety - > PEOU	−0.263	−0.269	0.038	6.711	0.109	0.000	Supported
Internet self-efficacy - > PU	0.041	0.065	0.035	1.923	0.008	0.055	Rejected
Internet self-efficacy - > PEOU	0.277	0.266	0.038	6.7	0.108	0.000	Supported
PU - > Attitude	0.538	0.538	0.049	11.369	0.378	0.000	Supported
PU - > Intention		0.375		7.552	0.233	0.000	Supported
PEOU - > PU	0.368	0.344	0.050	7.209	0.159	0.000	Supported
Perceived ease of use - > Attitude	0.303	0.308	0.049	6.467	0.124	0.000	Supported
Attitude - > Intention	0.620	0.541	0.047	10.778	0.485	0.000	Supported
Indirect effects							
Output quality - > PU		0.146		5.829		0.000	Supported
Output quality - > Attitude	0.519	0.522	0.031	17.182		0.000	Supported
Output quality - > Intention	0.454	0.556	0.029	20.527		0.000	Supported
Internet anxiety - > PU	−0.097	−0.093	0.020	4.8		0.000	Supported
Internet anxiety - > Attitude	−0.089	−0.086	0.029	2.97		0.003	Supported
Internet anxiety - > Intention	−0.137	−0.049	0.028	1.713		0.087	Rejected
Internet efficacy - > PU	0.102	0.092	0.019	4.985		0.000	Supported
Internet self-efficacy - > Attitude	0.161	0.166	0.026	6.34		0.000	Supported
Internet self-efficacy - > Intention	0.186	0.149	0.026	5.556		0.000	Supported
PU - > Intention		0.291		7.836		0.000	Supported
PEOU - > Attitude	0.198	0.185	0.031	6.457		0.000	Supported
Perceived ease of use - > Intention	0.311	0.396	0.034	9.904		0.000	Supported
Total effects							
Output quality - > PU	0.724	0.728	0.028	27.618		0.000	Supported
Output quality - > PEOU	0.424	0.425	0.036	11.006		0.000	Supported
Output quality - > Attitude	0.519	0.522	0.031	17.182		0.000	Supported
Output quality - > Intention	0.454	0.556	0.029	20.527		0.000	Supported
Internet anxiety - > PU	−0.018	−0.006	0.038	0.157		0.875	Rejected
Internet anxiety - > PEOU	−0.263	−0.269	0.038	6.711		0.000	Supported
Internet anxiety - > Attitude	−0.089	−0.086	0.029	2.97		0.003	Supported
Internet anxiety - > Intention	−0.137	−0.049	0.028	1.713		0.087	Rejected
Internet self-efficacy - > PU	−0.018	0.157	0.038	4.491		0.000	Supported
Internet self-efficacy - > PEOU	0.277	0.266	0.038	6.7		0.000	Supported
Internet self-efficacy - > Attitude	0.161	0.166	0.026	6.34		0.000	Supported
Internet self-efficacy - > Intention	0.186	0.149	0.026	5.556		0.000	Supported
PU - > Attitude	0.538	0.538	0.049	11.369		0.000	Supported
PU - > Intention	0.335	0.666	0.045	19.938		0.000	Supported
Perceived ease to use - > PU	0.368	0.344	0.050	7.209		0.000	Supported
Perceived ease to use - > Attitude	0.501	0.493	0.044	11.403		0.000	Supported
Perceived ease to use - > Intention	0.311	0.396	0.048	9.904		0.000	Supported
Attitude - > Intention	0.620	0.541	0.047	10.778		0.000	Supported

**Table 6:** Cross-validated redundancy of construct.

	*Q* ^2^
PU	0.527
PEOU	0.485
Attitude	0.60
Intention	0.59

The Cohen’s f2 method was used to calculate the size of the constructs’ effects. We examined the size of the proposed relationships between model constructs. Table 5 show the value of the path coefficients of the assumed relationships between the constructs and a summary of the hypotheses tested. The output quality variable was able to affect both the PU constructs (*β* = 0. 0.582, *f^2^* = 0.657, *t* = 15.205) and the PEOU constructs (*β* = 0.425, *f^2^* = 0.354, *t* = 11.006) at the significant level of *p* < 0.0001. The effect of output quality on PU and PEOU was thus found to be strong.

Internet anxiety also affected PU (*β* = 0.087, *f^2^* = 0.014, *t* = 2.481, *p* < 0.05) and PEOU (*β* = −0.269, *f^2^* = 0.109, *t* = 6.711, *p* < 0.0001), weakly and moderately, respectively. The effect of Internet anxiety on PU was positive, while on PEOU, it was negative.

The findings also showed that Internet self-efficacy affected PEOU (*β* = 0.266, *f^2^* = 0.108, *t* = 6.7, *p* < 0.0001), while having no effect on PU. PEOU, in turn, affected PU (*β* = 0.344, *f^2^* = 0.159, *t* = 7.209, *p* < 0.0001).

PU (*β* = 0.537, *f^2^* = 0.378, *t* = 11.369, *p* < 0.0001) and PEOU (*β* = 0.308, *f2* = 0.124, *t* = 6.467, *p* < 0.0001), respectively, can strongly and moderately influence attitude toward participating in online learning programs. Attitude (*β* = 0.541, *f^2^* = 0.485, *t* = 10.778, *p* < 0.0001) and PU (*β* = 0.375, *f^2^* = 0.233, *t* = 7.552, *p* < 0.0001) can predict the intention. Table 5 shows the direct, indirect, and total effect.

## 5. Discussion

This study confirms the application of TAM as a strong theoretical framework for understanding predictors of the use of online learning systems. This study also takes a critical step in further developing the TAM with the three interpretive constructs of output quality, Internet anxiety, and Internet self-efficacy.

The findings confirm that these three constructs can influence the attitude and willingness to use online education systems through perceived usefulness and perceived ease. The proposed research model will predict 62 and 74 percent of attitude and intention variance changes, respectively.

The results showed that the behavioral intention to use online learning in the future is directly explained by people’s attitudes toward using online learning and PU. Attitude was the strongest predictor of behavioral intention. Attitude is the reflection of people’s feelings and appraisals relating to a concept or subject. Here, attitude refers to one’s feelings about online learning systems. Students had greater intention to use online learning systems if they thought that doing so was a good idea and they felt positive about it.

Along with the research findings, several studies confirm the significant effect of attitude on the intention to use online learning (Lee et al., 2013; Calisir et al., 2014; Chang et al., 2017; Hussein, 2017; Hanif et al., 2018; Al-Rahmi et al., 2019; Buabeng-Andoh, 2021).

One of the factors that most affect attitude is PU: the results show the PU to strongly affect attitude. Phillips et al. (1994) show that PU reflects the psychological potential of end-users to consider using new technology as beneficial for personal and organizational well-being. In other words, when students find the use of online learning to be useful and beneficial, they develop a positive attitude toward using that system. Research confirms the significance and positive effect of PU on attitude (Huang, 2016; Briz-Ponce et al., 2017; Al-Rahmi et al., 2019; Revythi and Tselios, 2019; Salloum et al., 2019).

PEOU also has a positive effect on attitude, but this effect is not the same as that of PU— the effect size of PEOU on attitude was, in fact, moderate. This relationship has been confirmed in various studies (Huang, 2016; Briz-Ponce et al., 2017; Al-Rahmi et al., 2019; Revythi and Tselios, 2019; Salloum et al., 2019). Thus, PEOU and PU are shown to have directly influenced people’s attitudes toward using online learning systems. PEOU also indirectly affects attitude and intention. As PEOU measures the user-friendliness of a particular tool or method, the user will have a greater intention to use the tool if they consider that tool to be relatively user-friendly (Barat et al., 2009). The findings also show the importance of perceived convenience in enhancing the intention of users to use online learning systems through the moderating role of PU.

According to the findings, the PU predicted the intention to use online education systems, and the effect size was strong. If people expect the information and educational services provided by online systems to improve their learning, then they will likely be more inclined to use these systems. These results are consistent with many past studies (Calisir et al., 2014; Alqahtani et al., 2016; Tarhini et al., 2017; Fallah Haghighi et al., 2018; Al-Rahmi et al., 2019; Hariguna, 2019; Salloum et al., 2019; Tao et al., 2019). Our study is also consistent with Li et al. (2012) and Tao et al. (2019) who confirmed the indirect effect of PU on intention through attitude. The study by Park et al. (2012) on the acceptance of collaborative learning technologies also confirms this finding.

In line with past research we found that PEOU could strongly affect PU (Luarn and Lin, 2005; Lee, 2009; Park et al., 2012; Kim, 2014; Bhatiasevi and Yoopetch, 2015; Alqahtani et al., 2016; Chang et al., 2017; Ebrahimi et al., 2018; Joo et al., 2018; Tao et al., 2019). Students will find online learning useful and effective if they are comfortable using the Internet and the learning system and as long as they do not encounter any difficulties or problems with them.

Based on our findings, output quality and Internet self-efficacy had a significant indirect effect on intention in addition to the main TAM constructs. While Internet anxiety did not have a significant indirect effect on intention, it did have has a direct positive effect on PU. A review of studies shows there to be contradictions about the impact of Internet anxiety on PU. Zhang (2005) confirmed the positive effect of Internet anxiety on PU with respect to telecommunications employees using the Internet. Other research findings reported the negative effect of Internet anxiety on PU (Park et al., 2012; Purnomo and Lee, 2013).

Internet anxiety also had a negative effect on PEOU with a moderate effect size. The more students experience unrest, panic, fear, and anxiety in general when using the Internet, the more difficult it is to use a learning system. The negative effect of anxiety on PEOU has also been confirmed in research (Karaali et al., 2011; Chen and Tseng, 2012; Park et al., 2012; Ali et al., 2013; Calisir et al., 2014; Al-Gahtani, 2016).

The findings showed that output quality is a direct determinant of PU and PEOU. Perceptions of output quality results from cognitive instrumental processes (Venkatesh and Davis, 2000). In this study, determining output quality means comparing the result of learning online with the perceived quality of learning in traditional classrooms. In fact, students’ assessment of the quality of outcome of online classes may be different from the perceived output quality of learning in traditional classrooms. Tao et al. (2019) found that perceived quality affects the PU of massive open online courses. Consistent with our results, the positive effect of output quality on PU (Ahn et al., 2004; Vijayasarathy, 2004; Ahn et al., 2007) and on PEOU (Lee, 2010) was confirmed. Al-Nuaimi et al. (2022) also found that learning management technical system quality has demonstrated the most critical impact on the PU.

According to our findings, Internet self-efficacy indirectly affected attitudes and intention. The research of Isaac et al. (2017) found Internet self-efficacy to be a strong factor in the intention of employees in Yemeni government institutions to use online systems. However, the direct effect of self-efficacy on PU was only approaching significance (*p* = 0.055) while effect of self-efficacy on PEOU was moderate. Here, Internet self-efficacy refers to the belief of a person in their ability to use the Internet. In this regard, Tsai et al. (2014) showed that system self-efficacy can affect the PU and PEOU of telehealth systems. Similarly, Arpaci et al. (2019) found that self efficacy has direct effect on the PEOU.

Faqih (2013) examined consumer intention in Jordan and confirmed that the influence of Internet self-efficacy on PEOU was positive and that strong Internet self-efficacy affects PU positively with 99% confidence. Research also confirms the positive and strong effect of Internet self-efficacy on PU and PEOU (Park, 2009; Isaac et al., 2017).

## 6. Theoretical and practical implications

Theoretically, the current study provides new evidence to the existing literature supporting the TAM by confirming the significant positive effects of PU and PEOU on attitudes toward online learning systems. This study suggests that the intention to use online learning systems can be increased by developing the constructs of internet anxiety, internet effectiveness, and output quality.

The outcomes of the current study provide valuable knowledge and offer a deeper understanding of external factors and provide useful practical suggestions for policymakers, professionals, developers, and designers in the effective use of online learning systems during and beyond Covid-19. The findings may have implications for organizations planning to design and implement online learning systems.

As PU is a direct predictor of behavioral intention, managers can use this to enhance people’s beliefs about how online learning systems can improve their learning performance and thereby increase the intention of students to use them even in non-emergency health situations. Specifically, students need to be made aware of how online learning systems will benefit them and improve their performance. In this research, PU has been shown with criteria such as the value of the online learning experience, the objectivity of the online learning results, and the minimizing of educational inequality. Managers should review communication materials (e.g., websites and brochures) designed to publicize these systems and make sure the utility in individual level such as increased learning and social benefits of online learning such as reducing educational is emphasized.

As attitude was the most important predictor of intention, and PU and PEOU both directly and indirectly affected intention, educators need to ensure that online learning is beneficial to improving student learning outcomes. Superior teaching techniques should provide for greater success. The findings imply administrators and online educators must devote resources to ensuring online education is designed appropriately and effectively. It is not enough to simply place an established class online; online courses need to be designed using best practices for this type of educational modality.

Considering the effect of output quality on PU and PEOU, there should be a statistical comparison of the learning performance of online and traditional learning systems. This analysis can also ensure quality of online learning offerings. Additionally, these findings can be also shared with students to help them make decisions regarding the usefulness of online learning. Providing information in this area can improve people’s understanding of learning quality. The use of user-friendly methods *via* a variety of educational techniques, including videos, photos, text news, etc. seems to improve people’s understanding of learning quality. Moreover, given the negative impact of Internet anxiety on PEOU, students need to be specifically educated on Internet use. Educational institutions offering online learning should consider offering seminar type courses to improve comfort with Internet use, or embedding this type of training into the beginning of online courses.

This study provides a strong insight for the government and the Ministry of Education to provide all the educational material related to the internet and computers to the students. For this purpose, it is suggested that students be provided with the purchase of powerful modems and wireless and high-speed internet packages on Sim-cards.

The findings strongly suggest, however, that online learning systems should feature ease of use and simplify the learning process. Therefore, it is important to improve features of online learning system, helping users complete tasks with the less time and effort. Efforts should be made to guarantee that online learning systems are of a simple yet advanced design that allow the inclusion of a variety of educational methods. Therefore, it is recommended to use easy and user-friendly platforms with the ability to share all kinds of files and videos, with the ability to provide interactive presentations in the simplest possible mode, for training.

Perceived quality output also has an influence on the intent to usage online education. The disadvantages of unexpected operation of online education could lead government, regulators and other practitioners to advance the online education as well as increase the stdent leaning. Clearly, respected criticism from learner and educator should be considered for future improvment since the learning methods have extremely reformed due to the Covid-19 pandamic. The criticisms could inspire the educational institute to improve the quality of platform.

## 7. Conclusion and limitations

This study has several important conclusions: First, PU and PEOU must be taken seriously in order to bring a successful online learning system into effect. Students’ attitudes to using online learning systems were the strongest predictors of behavioral intention. The findings confirm the direct and indirect impact of output quality on all endogenous constructs including PU, PEOU, attitude, and intention. As university administrators need to improve the learning performance of individuals, output quality needs to be made an urgent priority in learning system projects. If users perceive that virtual learning systems will enhance learning performance to a greater extent than traditional learning systems do, they will have higher PU and therefore look more favorably on online learning systems and be more inclined to use them. Output quality was the strongest predictor of PU and PEOU. Educational planners and designers thus need to pay adequate attention to this important element.

The present study, like other studies, has limitations that need to be considered when this topic is tackled in the future. First, this study was conducted with agricultural students at a limited number of universities in Iran, where practical and experimental courses were lacking, and in which various online education systems were used. Our findings were extracted from the analysis of this data. Generalizations to other students and other universities should thus be made with caution.

Second, this study integrated the three key constructs of output quality, Internet anxiety, and Internet self-efficacy into TAM. Further studies have shown that other constructs can be used to improve the prediction of the intention to use online learning. Which suggests additional variables could be added in future studies.

One of the important limitations of this study was how the questions were assessed. In this study, items were measured as self-reported. To address the potential influence of common method bias we used a range of different items to measure our sample perception. Furtherrmore we used positive and negative (Reverse items) items and finally adapting our measures from reliable and valid instruments helps us in this regard. However, it is suggested, that other methods of data collection, such as interviews and focus groups, be used in other studies beside structured questionnaires.

This research was also cross-sectional and evaluated people’s perceptions and intention at a specific time, and particularly during the peak of a global pandemic. People’s perceptions will, however, change over time, as their experience increases (Venkatesh et al., 2003). Future researchers are advised to consider using longitudinal studies.

## Data availability statement

The original contributions presented in the study are included in the article/Supplementary material, further inquiries can be directed to the corresponding author/s.

## Ethics statement

Ethical review and approval was not required for the study on human participants in accordance with the local legislation and institutional requirements. All procedures performed in studies involving human participants were in accordance with the ethical standards of the institutional and/or national research committee and with the 1964 Helsinki declaration and its later amendments or comparable ethical standards. Written informed consent was obtained from all subjects involved in the study.

## Author contributions

TZ: conceptualization, methodology, software, formal analysis, investigation, writing – original draft, and writing – review and editing. SH: methodology, software, validation, formal analysis, writing – original draft, and writing – review and editing. MY: conceptualization, methodology, software, validation, formal analysis, investigation, writing – original draft, writing – review and editing, visualization, and supervision. NK and LW: formal analysis, writing – review and editing. All authors contributed to the article and approved the submitted version.

## Conflict of interest

The authors declare that the research was conducted in the absence of any commercial or financial relationships that could be construed as a potential conflict of interest.

## Publisher’s note

All claims expressed in this article are solely those of the authors and do not necessarily represent those of their affiliated organizations, or those of the publisher, the editors and the reviewers. Any product that may be evaluated in this article, or claim that may be made by its manufacturer, is not guaranteed or endorsed by the publisher.
